# Rapid urinary lipoarabinomannan test with laboratory-level sensitivity for tuberculosis detection: a performance evaluation

**DOI:** 10.1128/spectrum.03042-25

**Published:** 2025-12-31

**Authors:** Qisheng Jiang, Harisha Ramachandraiah, Carolyn Duncan, Sumanth Gandra, Ige A. George, Jingyi Luan, Marcos Perez, Lorraine Lillis, David S. Boyle, Scott Crick, Morten Ruhwald, Srikanth Singamaneni

**Affiliations:** 1 Brightest Bio, Saint Louis, Missouri, USA; 2 FIND, the global alliance for diagnostics 91635, Geneva, Switzerland; 3 Division of Infectious Diseases, Department of Medicine, Washington University School of Medicine 12275 , St. Louis, Missouri, USA; 4 Department of Mechanical and Aerospace Engineering, University of Houston 14743, Houston, Texas, USA; 5 PATH, Seattle, Washington, USA; 6 Department of Mechanical Engineering and Materials Science, Institute of Materials Science and Engineering, Washington University in St. Louis 685419, St. Louis, Missouri, USA; ICON plc, London, United Kingdom

**Keywords:** tuberculosis, lipoarabinomannan, rapid diagnostic test, plasmonic fluor, diagnostic accuracy

## Abstract

Developing rapid, non-sputum-based tests is a global priority to improve the diagnosis and timely treatment of tuberculosis (TB). In this study, we evaluated the detection limit, precision, stability, and lot-to-lot variation of a next-generation rapid diagnostic test (RDT), the plasmonic fluor (PF)-enhanced urinary lipoarabinomannan lateral flow assay (PF-LAM). We also assessed the diagnostic performance of PF-LAM, Alere Determine TB LAM Ag (AlereLAM), and electrochemiluminescence LAM assay (EclLAM), a highly sensitive laboratory-based test by comparing with microbiology reference standard (MRS) that includes molecular testing (Xpert MTB/RIF) and culture. Two sub-studies were conducted using banked urine samples. First, a preclinical study involving 399 well-characterized urine samples was conducted to determine assay cutoff and assess PF-LAM performance compared with Alere LAM. Second, a diagnostic accuracy assessment study was conducted using 77 blinded samples to compare the performance of PF-LAM to EclLAM. In the preclinical study, when compared with MRS, the AlereLAM showed a sensitivity of 5.5% (95% CI: 3.1%–9.6%) and a specificity of 98.5% (95% CI: 95.6%–99.6%), while PF-LAM exhibited a sensitivity of 33.8% (95% CI: 27.4%–40.6%) and the same specificity of 98.5% (95% CI: 95.6%–99.6%). In the diagnostic accuracy study, when compared with MRS, PF-LAM achieved a sensitivity of 58% (95% CI: 41%–73%) and a specificity of 98% (95% CI: 88%–100%), while EclLAM showed a sensitivity of 42% (95% CI: 27%–59%) and a specificity of 95% (95% CI: 85%–99%). Overall, PF-LAM demonstrated better analytical and diagnostic sensitivity compared with EclLAM and significantly better performance compared with AlereLAM, while exhibiting high precision and minimal lot-to-lot variability.

**IMPORTANCE:**

To the best of our knowledge, this study uniquely reports a rapid diagnostic test, plasmonic fluor-enhanced urinary lipoarabinomannan lateral flow assay (PF-LAM), outperforming an electrochemiluminescence-based laboratory assay. PF-LAM demonstrated promising analytical and diagnostic performance with minimal lot-to-lot variability, positioning it as a helpful tool for non-sputum tuberculosis diagnostics.

## INTRODUCTION

Tuberculosis (TB) remains a leading infectious cause of death globally, with over a third of new TB cases (about 3 million annually) undiagnosed and unreported to the health system (1). This diagnostic gap stems from the suboptimal yield of current pulmonary TB tests (sputum smear/culture, molecular assays, and chest radiography) and their limited applicability in point-of-care (POC) settings (2). Developing rapid non-sputum tests is therefore a top priority to accelerate diagnosis and treatment (3).

Urinary lipoarabinomannan (uLAM) has emerged as a promising biomarker for non-sputum TB diagnostics (4). The Determine TB LAM Ag test (AlereLAM, Abbott, USA) is the only WHO-recommended rapid diagnostic test (RDT) for uLAM, but its sensitivity is suboptimal, particularly in HIV-negative individuals or those with well-controlled HIV (85% of TB patients) (5–7).

A laboratory-based research-use-only LAM assay using electrochemiluminescence (EclLAM, Meso Scale Diagnostics, USA) shows markedly higher sensitivity than AlereLAM and other RDTs and is widely regarded as the sensitivity benchmark for uLAM detection (8–10). Studies suggest that new RDTs must match or exceed the sensitivity of EclLAM to achieve the WHO’s target product profile (TPP) rapid TB detection. Achieving laboratory-level performance in a rapid format is challenging due to the sensitivity limits of conventional optical labels, often requiring signal amplification or sample preconcentration. Despite progress, there remains a critical need for a simple, robust, and highly sensitive next-generation RDT for uLAM (11–16). In addition to sensitivity, lot-to-lot variability must also be addressed before large-scale field use (17).

Here, we demonstrate a next-generation, ultra-sensitive, urine-based LAM lateral flow assay with minimal lot-to-lot variability. The assay leverages an ultra-bright fluorescent label, plasmonic fluor (PF), which is 7,000-fold brighter compared with conventional molecular fluorophores (18). This innovation improves the signal-to-noise ratio by 1,000-fold and significantly boosts analytical and clinical sensitivity. PFs have been successfully applied in various assay formats, including lateral flow assays (LFAs), for detecting a wide range of analytes (19–21). Through extensive optimization using a pair of high-affinity monoclonal antibodies, we have developed an ultra-sensitive PF-enhanced lateral flow assay for detection of TB LAM in urine (PF-LAM). In this study, we aimed to evaluate its analytical performance, precision and stability, and the diagnostic performance compared to EclLAM and AlereLAM using microbiology reference standard (MRS) that includes molecular testing (Xpert MTB/RIF) and culture among banked urine samples.

## RESULTS

### PF-LAM workflow and assay detection limit

**Fig 1:**
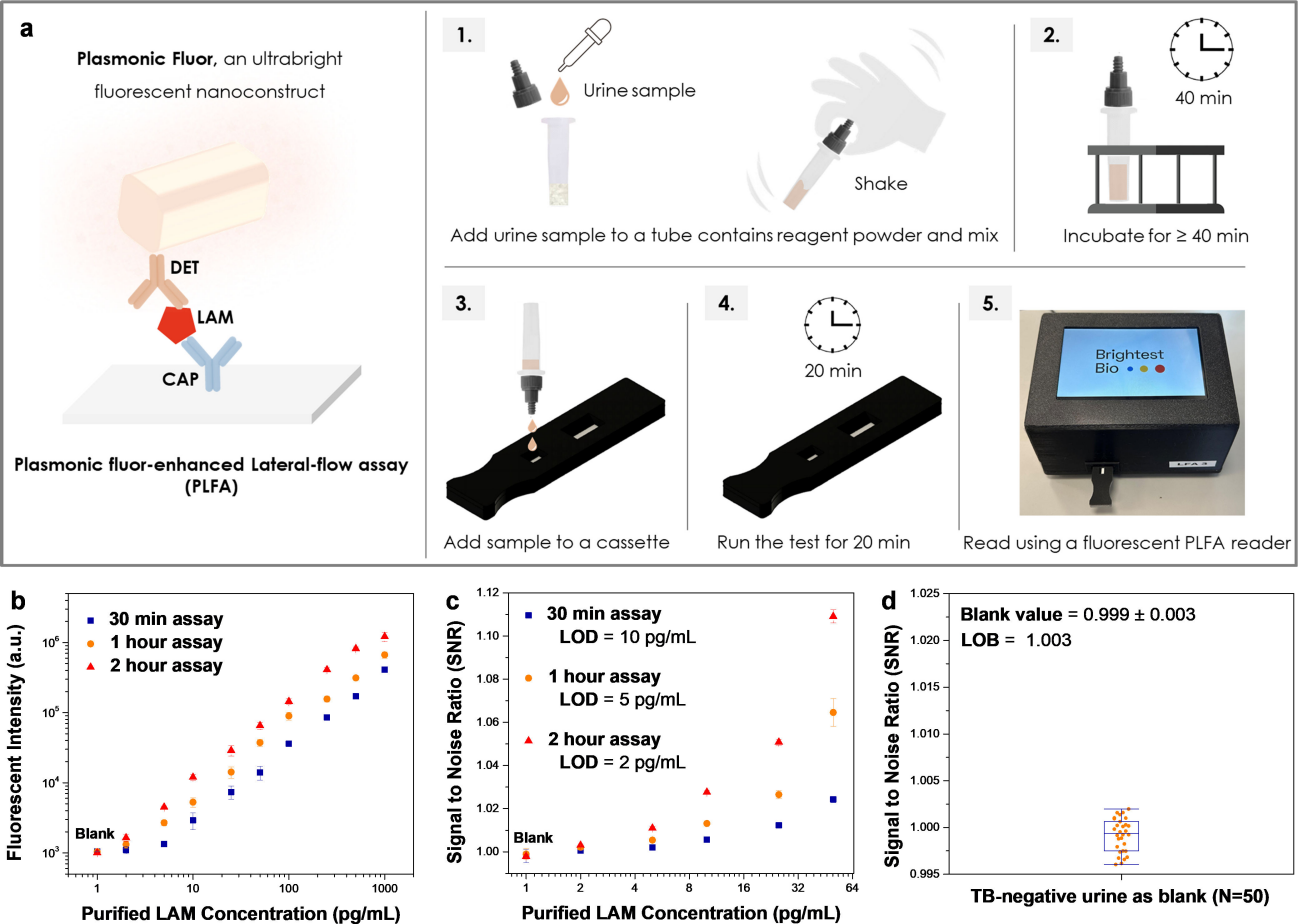
PF-LAM workflow and detection limit. (**a**) PF-LAM principle and workflow. (**b**) Dose-response fluorescent intensities of PF-LAM generated by spiking purified cLAM in TB-negative urine with different assay time. (**c**) Dose-response signal to noise ratio of PF-LAM generated by spiking purified cLAM in TB-negative urine with different assay times. (**d**) SNR of PF-LAM tested with TB-negative samples for the determination of LOB (*N* = 50). PF-LAM is a plasmonic fluor-enhanced lateral flow assay. LAM, lipoarabinomannan; CAP, capture antibody; DET, detection antibody.

The workflow of PF-LAM assay is illustrated in Fig. 1a. By varying the sample incubation time (10-, 40-, and 100-min), the total assay durations can be adjusted to 30 min, 1 h, and 2 h, respectively. Analytical performance was evaluated using urine samples spiked with cultured LAM (cLAM). For the 30-min assay (10-min incubation and 20-min running time), PF-LAM achieved a LOD of 10 pg/mL. Increasing the incubation time to 40- and 100-min, further enhanced analytical sensitivity, improving the LOD to 5 and 2 pg/mL, respectively (Fig. 1b and c). The details of analytical sensitivity panel concentration, fluorescent intensity, and signal-to-noise ratio (SNR) are shown in Tables S1 and S2. PF-LAM demonstrated a blank SNR ratio of 0.999 ± 0.003, resulting in a limit of blank (LOB) of 1.003 for testing TB-negative urines.

### Precision and stability of PF-LAM

**Fig 2:**
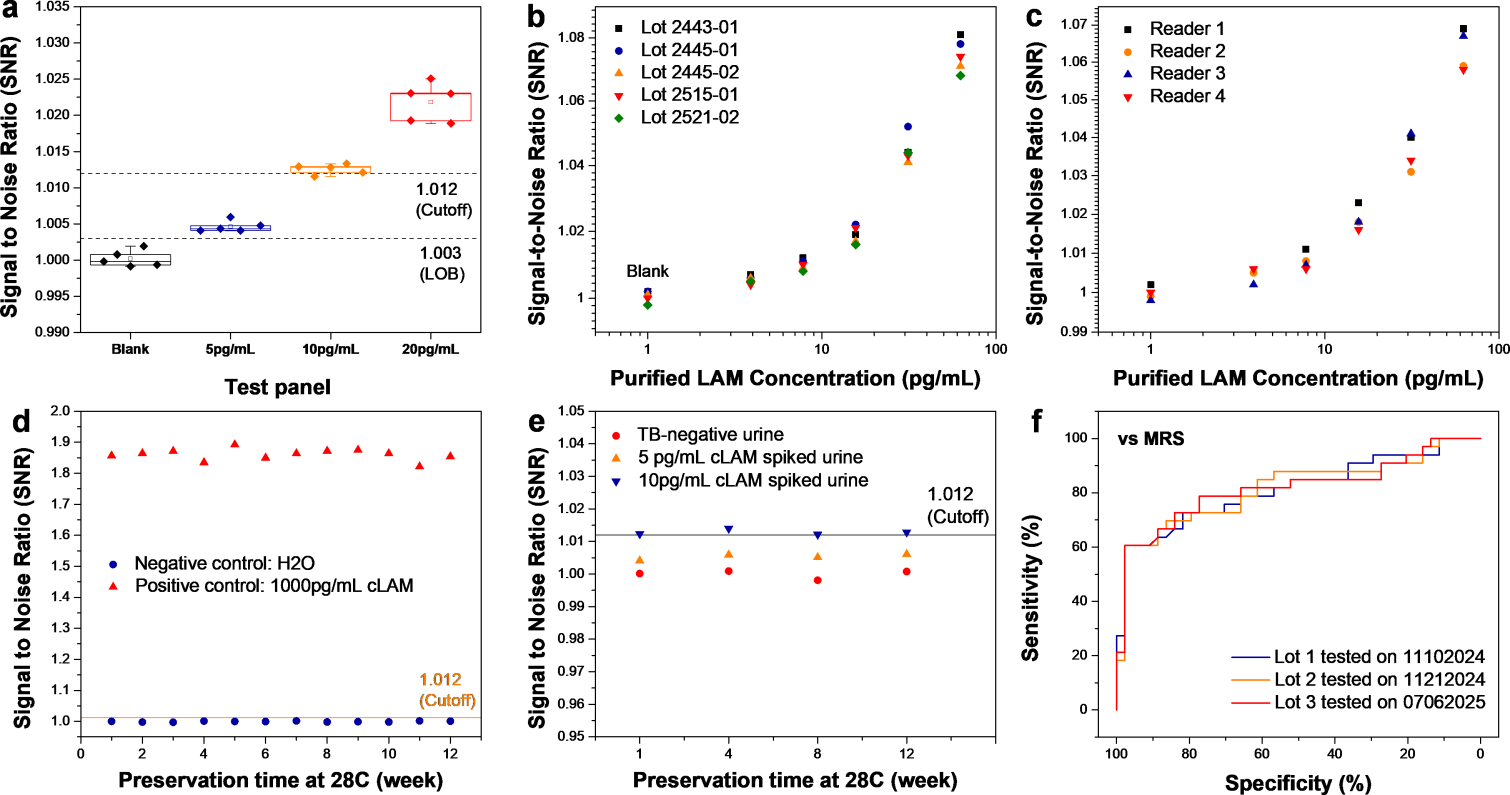
Precision and stability of PF-LAM. (**a**) Repeatability and reproducibility panel (TB-negative urine as blank, 5 pg/mL cLAM-spiked urine, 10 pg/mL cLAM-spiked urine, and 20 pg/mL cLAM-spiked urine) tested by PF-LAM. (**b**) Dose-response SNR of PF-LAM generated by five different lots. (**c**) Dose-response signal to ratio of PF-LAM generated by four different readers. (**d**) Stability test of PF-LAM using negative control: H_2_O and positive control: 1,000 pg/mL cLAM in H_2_O under 28°C preservation. (**e**) Stability test of PF-LAM using TB-negative urine, 5 pg/mL cLAM-spiked urine, and 10 pg/mL spiked cLAM under 28°C preservation. (**f**) ROC curves of PF-LAM data obtained on different days using different lots on the diagnostic accuracy panel.

Figure 2a shows PF-LAM repeatability with consistent results across TB-negative urine and cLAM-spiked urine at 5 and 10 pg/mL, concentrations near the LOB and assay cut-off. A dose-response panel (3.9–62.5 pg/mL cLAM) was evaluated with five PF-LAM kit lots, producing reproducible results (Fig. 2b; Table S3). One test was scanned on four different LFA readers, yielding highly consistent signals (Fig. 2c; Table S4).

For stability, PF-LAM kits stored at 28°C for 12 weeks maintained performance. As shown in Fig. 2d and e, SNRs for negative control (H_₂_O), positive control (1,000 pg/mL), TB-negative urine, and 5–10 pg/mL cLAM spiked urine remained stable over time.

Figure 2f shows the comparison of ROC curves of the PF-LAM assay performance on the diagnostic panel conducted on different days using multiple lots of test plates. The PF-LAM tests performed on different days using different lots of kit showed identical sensitivity and specificity.

### Preclinical study: cut-off determination and performance comparison with AlereLAM

**Fig 3:**
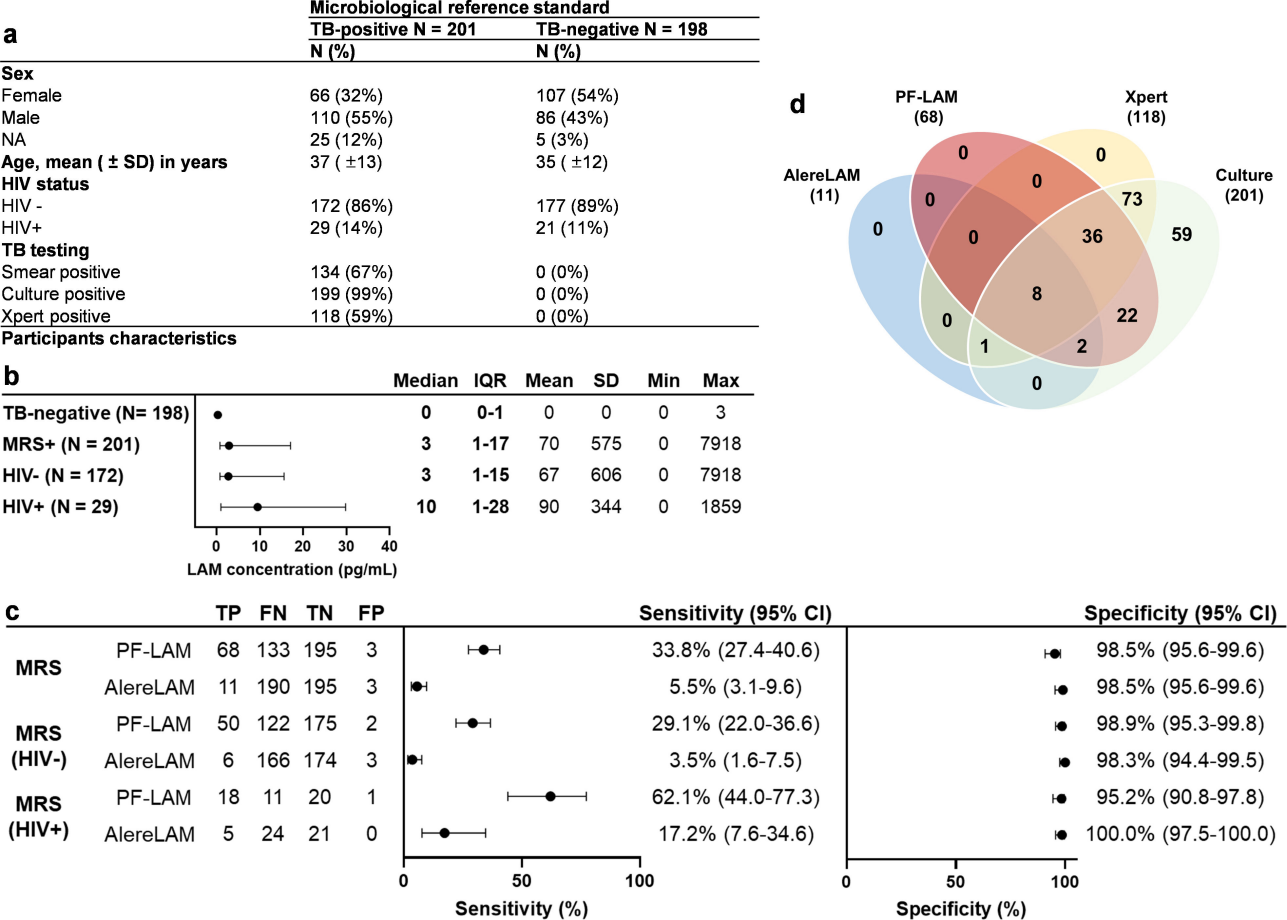
Preclinical study compared with AlereLAM. (**a**) Participants’ characteristics at the time of urine collection in the preclinical panel. (**b**) LAM concentration (pg/mL) in TB-negative and TB-positive samples from the preclinical panel (plot showing median/IQR). (**c**) Head-to-head diagnostic accuracy comparison of PF-LAM and AlereLAM vs MRS and stratified by HIV statuses. (**d**) Venn diagram of PF-LAM, AlereLAM, and Xpert positivity among the 201 TB-positive cases in the preclinical panel. MRS, microbiological reference standard; TP, true positive; FN, false negative; TN, true negative; FP, false positive.

In this preclinical study, 399 urine samples were tested, including 201 TB-positive and 198 TB-negative cases (confirmed by smear, Xpert, and culture) (Fig. 3a). Median uLAM concentrations were 0 pg/mL (IQR 0–1) in TB-negative samples and higher in TB-positive cases: 3 pg/mL overall (IQR 1–17), 3 pg/mL in HIV-negative (IQR 1–15), and 10 pg/mL in HIV-positive samples (IQR 1–28) (Fig. 3b).

The specificities of Alere LAM and PF-LAM (with the optimal cut-off, Fig. S1; Table S5) were both 98.5% (195/198, 95% CI: 95.6%–99.6%) compared with the microbiological reference standard (MRS). When stratified by the HIV status, the specificities of AlereLAM and PF-LAM were 98.3% (174/177, 95% CI: 94.4%–99.5%) and 98.9% (175/177, 95% CI: 95.3%–99.8%) for HIV-negative samples; 100.0% (21/21, 95% CI: 97.5%–100.0%) and 95.2% (20/21, 95% CI: 90.8%–97.8%) for HIV-positive samples.

Using the MRS, AlereLAM showed a sensitivity of 5.5% (11/201, 95% CI: 3.1%–9.6%), while PF-LAM reached 33.8% (68/201, 95% CI: 27.4%–40.6%, Fig. 3c). In HIV-negative samples, sensitivities were 3.5% (6/172, 95% CI: 1.6%–7.5%) for AlereLAM and 29.1% (50/172, 95% CI: 22.0%–36.6%) for PF-LAM. Among HIV-positive cases, AlereLAM had a sensitivity of 17.2% (5/29, 95% CI: 7.6%–34.6%) versus 62.1% (18/29, 95% CI: 44.0%–77.3%) for PF-LAM. Across all comparisons, the lower bound of PF-LAM’s 95% CI exceeded the upper bound of AlereLAM’s, with differences of 17.8% (MRS), 14.5% (HIV-negative), and 9.4% (HIV-positive). A performance comparison of PF-LAM and AlereLAM on HIV-positive cases stratified by CD4 count is provided in Table S6. Overall, PF-LAM identified 6.2 times more TB-positive cases than AlereLAM (68 vs 11, Fig. 3d).

### Diagnostic accuracy assessment of PF-LAM compared with EclLAM

**Fig 4:**
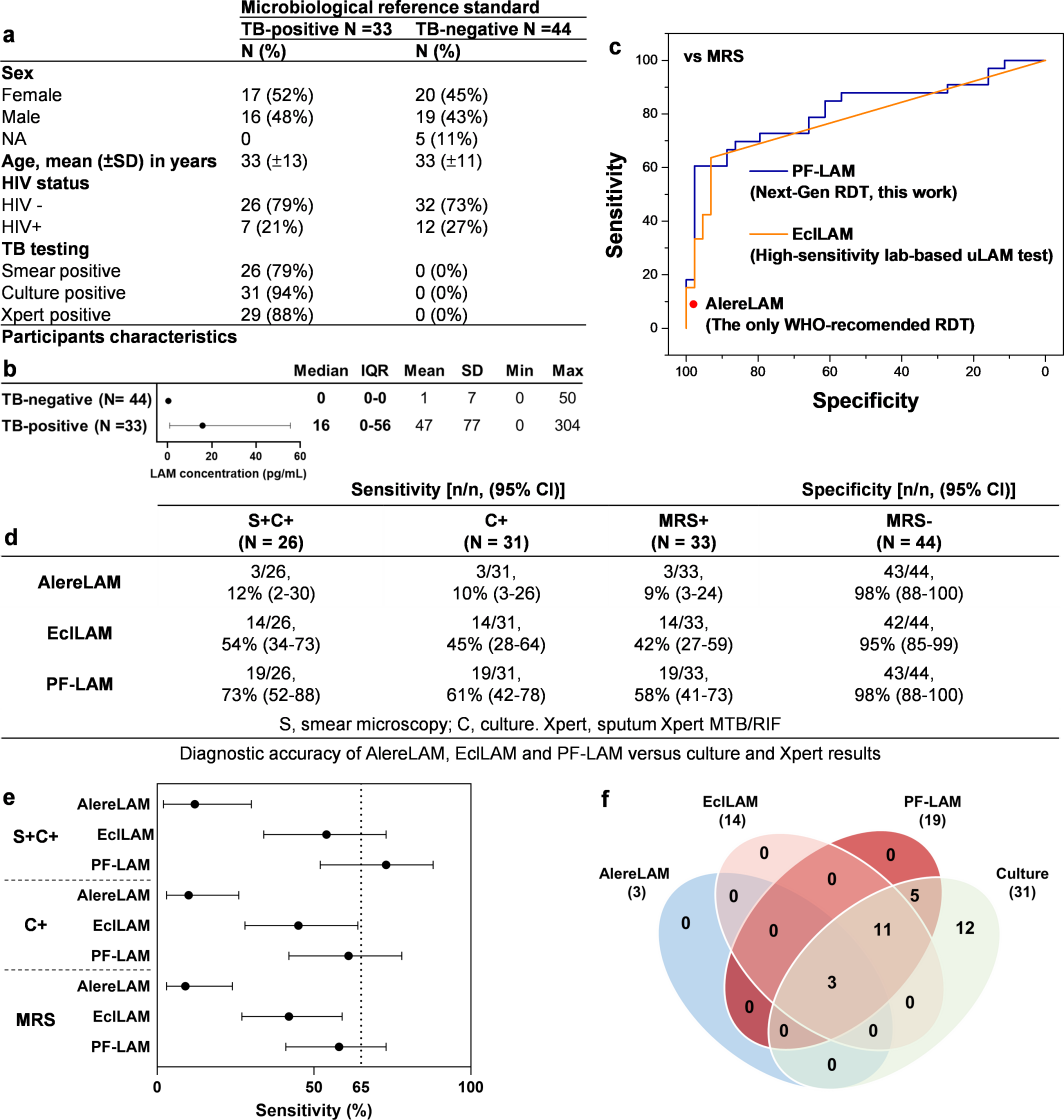
Diagnostic accuracy study compared with EclLAM and AlereLAM. (**a**) Participants’ characteristics at the time of urine collection in the diagnostic accuracy panel. (**b**) LAM concentration (pg/mL) in TB-negative and TB-positive samples from the diagnostic accuracy panel (plot showing median/IQR). (**c**) Receiver operating characteristic (ROC) curves of EclLAM (PATH) and PF-LAM in the diagnostic accuracy assessment study. (**d**) Diagnostic accuracy results of AlereLAM, EclLAM, and PF-LAM versus culture and Xpert. (**e**) Head-to-head diagnostic accuracy comparison of PF-LAM, EclLAM, and AlereLAM. (**f**) Venn diagram of PF-LAM, EclLAM, and AlereLAM positivity among the 33 TB-positive cases in the diagnostic accuracy panel. MRS, microbiological reference standards; IQR, interquartile range; SD, standard deviation; Min, minimum; Max, maximum.

Among 77 urine samples in the blinded panel, 33 samples were from TB-positive, and 44 were from TB-negative patients (Fig. 4a). For the TB-negative samples, the median uLAM concentration measured by PFLISA-LAM was 0 pg/mL (IQR 0–0). In the TB-positive samples, the median uLAM concentrations were 16 pg/mL (IQR 0–56) (Fig. 4b; Tables S7 and S8).

Figure 4c compares ROC curves of the EclLAM (PATH) and PF-LAM assays, with PF-LAM showing an AUC of 0.82 (95% CI: 0.72–0.93). EclLAM results from MSD and PATH had identical sensitivity and specificity versus smear, culture, and Xpert (Fig. S2; Table S9). Among smear- and culture-positive (S+C+) cases, the sensitivities were 12% (3/26; 95% CI: 2%–30%) for AlereLAM, 54% (14/26; 95% CI: 34%–73%) for EclLAM, and 73% (19/26; 95% CI: 52%–88%) for PF-LAM (Fig. 4d). In culture-positive (C+) cases, the sensitivities were 10% (3/31; 95% CI: 3%–26%), 45% (14/31; 95% CI: 28%–64%), and 61% (19/31; 95% CI: 42%–78%) for AlereLAM, EclLAM, and PF-LAM, respectively. For MRS-positive cases, AlereLAM reached 9% (3/33; 95% CI: 3%–24%), EclLAM 42% (14/33; 95% CI: 27%–59%), and PF-LAM 58% (19/33; 95% CI: 41%–73%). The specificities were 98% (43/44; 95% CI: 88%–100%) for AlereLAM and PF-LAM, and 95% (42/44; 95% CI: 85%–99%) for EclLAM. Tables S10 and S11 summarize the performance of AlereLAM, PF-LAM, and EclLAM stratified by HIV status and CD4 count.

In all comparisons, PF-LAM and EclLAM showed significantly higher sensitivity than AlereLAM, with non-overlapping 95% CIs (Fig. 4e). The lower bound of the PF-LAM’s 95% CIs exceeded the upper bound of the AlereLAM by 22% for S+C+, 16% for C+, and 17% for MRS-positive cases. The lower bound of the EclLAM’s 95% CIs also surpassed the upper bound of the AlereLAM, with smaller differences: 4% for S+C+, 2% for C+, and 3% for MRS-positive cases. PF-LAM consistently demonstrated higher point estimates than EclLAM, with its lower bound closely approaching EclLAM’s point estimates and its upper bound near EclLAM’s highest values.

Overall, PF-LAM identified 6.3 times more TB-positive cases (19 vs 3) compared with AlereLAM and 1.4 times more cases (19 vs 14) compared with EclLAM (Fig. 4f).

Figure S3 shows ROC curves for PF-LAM at 30-, 60-, and 120-min assay times. Against MRS, sensitivity increased from 42% (14/33; 95% CI: 27%–59%) at 30 min to 61% (20/33; 95% CI: 44%–75%) at 2 h; against culture, from 45% (14/31; 95% CI: 28%–64%) to 65% (20/31; 95% CI: 45%–81%) (Table S12). Specificity remained 98% (43/44; 95% CI: 88%–100%) across all times. Importantly, PF-LAM at 30 min matched the sensitivity of EclLAM, which requires 2 h.

## DISCUSSION

Over recent years, traditional RDTs have been enhanced through the use of optimized antibodies, signal amplification, and sample preconcentration (11, 22). However, none of these RDTs have achieved the sensitivity level of laboratory-based EclLAM, which is considered the benchmark for meeting the WHO TPP sensitivity target for non-sputum-based TB tests. This study demonstrates that PF-LAM outperforms EclLAM in both analytical and diagnostic performance and shows significantly better performance compared with AlereLAM.

Previous studies have shown that EclLAM has a LOD range from 3 to 63 pg/mL (based on different plates) without a sample preconcentration step (23, 24). One study showed combining EclLAM with a preconcentration step (concentration factor of 7) can achieve a threshold around 5.2 pg/mL (10). It has been estimated that a cutoff of 5 pg/mL LAM or below is required to meet the TPP sensitivity target (6).

PF-LAM achieved a LOD of 10 pg/mL (30-min assay), 5 pg/mL (1-h assay), and 2 pg/mL (2-h assay) when detecting purified LAM in urine. The improvement in analytical sensitivity with elongated sample incubation time also translates to better diagnostic sensitivity. The sensitivity of PF-LAM increased from 45% to 61% and 65% (on the diagnostic panel versus culture) as the assay time increased from 30 min to 1 and 2 h. Importantly, while longer assay times enhance sensitivity, they do not negatively impact specificity of PF-LAM.

In this regard, PF-LAM offers flexibility and the option for sensitivity improvement by simply increasing the assay time from 1 to 2 h. The WHO-recommended rapid TB test should have a time-to-result of less than 1 h, as outlined in the recently published non-sputum rapid test target product profile (TPP). However, it is also noted that the ideal time-to-result has not been studied and may vary significantly between countries and settings. Therefore, the optimal assay time for PF-LAM should be further studied and determined in real-world clinical settings, with a focus on diagnostic yield.

Recent findings on the lot-to-lot variability of FujiLAM have raised important questions about the manufacturing, verification, and validation of RDTs, especially high-sensitivity RDTs (17). Besides technical optimization, quality control and precision evaluation based on the Technical specifications series for submission to WHO prequalification—diagnostic assessment (TSS) and Clinical and Laboratory Standards (CLSI)—are essential to ensure reliability. We have conducted preliminary precision and stability tests on PF-LAM, which showed good repeatability and reproducibility when measuring TB-negative urine and cLAM spiked urine samples near the LOB and assay cut-off. PF-LAM also demonstrated minimal lot-to-lot and reader-to-reader variability. In terms of stability, the PF-LAM kits showed highly stable performance over 12 weeks at 28°C. Moreover, three different lots tested on the same urine panel across more than six months yielded highly consistent results. These findings are encouraging but limited: (i) repeatability and reproducibility were only tested at one site; (ii) stability testing needs longer duration and higher stress conditions to define shelf-life. More extensive, multi-site studies of precision, lot variability, and stability are needed, and design and quality controls should be continuously refined.

In the preclinical study, AlereLAM demonstrated a sensitivity of 5.5% versus MRS and 17.2% among PLHIV, which is notably lower than values reported in the literature (10.8% for HIV-negative patients and 42% for PLHIV) (10, 11). Several factors may account for this discrepancy: (i) differences in the study population, (ii) variations in the sample collection procedures, and (iii) the use of frozen samples with prolonged storage times. Collectively, these factors could have contributed to the reduced performance of AlereLAM observed in this study. PF-LAM detected 68 positives versus 11 for AlereLAM (6.2-fold more) against MRS. Among HIV-negative participants, it detected 50 versus 6 (8.3-fold), and among HIV-positive 18 versus 5 (3.6-fold). Prior studies showed FujiLAM and EclLAM (with preconcentration) detected 4.9- and 6.2-fold more positives, respectively, in HIV-negative participants compared with AlereLAM, while FujiLAM detected 1.7-fold more in HIV-positive (Table S13) (5, 10). One limitation of this sub-study was that testing was unblinded; however, PF-LAM results were generated by the LFA reader algorithm, minimizing bias.

In the diagnostic accuracy study, PF-LAM showed better performance compared with EclLAM and AlereLAM. PF-LAM and EclLAM detected 6.3-fold (19 vs 3) and 4.7-fold (14 vs 3) more TB-positive cases than AlereLAM, respectively. Across both sub-studies, PF-LAM consistently detected at least six times more positive cases than AlereLAM.

PF-LAM demonstrated better analytical and diagnostic sensitivity than EclLAM, a 2-h, plate-based immunoassay requiring laboratory equipment and specialized skills. Notably, PF-LAM uses the same antibodies as the EclLAM but operates without specialized instruments or complex protocols (23). The boost of signal-to-noise ratio introduced by PF is likely the key factor that enabled PF-LAM to outperform EclLAM. This is highly encouraging, as no other RDT has matched, let alone exceeded, EclLAM’s performance. A limitation of this sub-study is the small sample size, resulting in wide sensitivity 95% CIs; while PF-LAM consistently showed higher point estimates, overlapping 95% CIs suggest that the differences may not be statistically significant. Future studies with larger sample sizes might be needed to confirm a definitive difference. A key strength of this sub-study was its rigorous, blinded design directly comparing the diagnostic accuracy of PF-LAM, EclLAM, and AlereLAM.

The main limitation of our study is the use of frozen urine samples, with ~88% (398/451) stored in the FIND biospecimen bank for 5–9 years (2016–2020). Prolonged storage and freeze–thaw cycles can markedly reduce measurable urinary LAM; one study reported a 50% loss after just one day at –70°C (25). Such degradation may have affected the diagnostic accuracy of PF-LAM, AlereLAM, and EclLAM. Future evaluations using fresh samples are needed to better reflect real-world performance. Another limitation of our study is the limited sample size for HIV-positive cases (15%, 36/234 among TB-positive cases, and 14%, 33/242 among TB-negative cases), which restricts our ability to fully evaluate the performance of PF-LAM in HIV-positive TB cases. We observed an increase in sensitivity and a decrease in specificity for PF-LAM among HIV-positive participants; however, given the limited number of HIV-positive cases in this study, further investigations with larger HIV-positive cohorts are needed to confirm these findings.

In summary, this study has demonstrated the development and performance evaluation of a next-generation, ultra-sensitive uLAM RDT (PF-LAM) with better analytical and diagnostic performance compared with EclLAM (high-sensitivity lab-based uLAM test) and AlereLAM (the only WHO-recommended RDT for uLAM). The findings support the need for larger prospective studies to thoroughly characterize the diagnostic accuracy of PF-LAM in diverse clinical settings. PF-LAM shows promise in potentially meeting the WHO’s TPP for a non-sputum TB diagnostic.

## MATERIALS AND METHODS

### Standard

Based on the WHO technical specifications series for rapid diagnostic tests to detect mycobacterial LAM antigen in urine (TSS-23) (26), the MRS for pulmonary TB-positive in this study was defined as positive result from either sputum molecular testing (Xpert MTB/RIF) or sputum liquid culture. Cases were classified as “TB-negative” if both molecular testing and culture of sputum specimens yielded negative results.

### Study design

To evaluate the performance of the PF-LAM assay, we conducted two sub-studies using banked urine samples:

1. Preclinical study: The TB team at the Foundation for Innovative New Diagnostics (FIND) initiated a preclinical evaluation using a well-characterized panel of 399 urine samples with known microbiological testing results, sourced from the FIND biospecimen bank. This study, conducted at Brightest Bio (Saint Louis, USA), aimed to determine the PF-LAM assay cut-off and evaluate its performance in comparison to AlereLAM using MRS as reference test.
2. Diagnostic accuracy assessment study: Following the preclinical findings, FIND commissioned a diagnostic accuracy study using a blinded panel of 77 urine samples from the FIND biospecimen bank. To have a consistent and accurate comparison, frozen aliquots of these samples were distributed to three testing sites: Meso Scale Diagnostics (MSD, Maryland, USA) and PATH (Seattle, USA) for evaluation using the EclLAM assay, and Brightest Bio for testing with both PF-LAM and AlereLAM. All test results were submitted to FIND, which then unblinded the panel data to facilitate comparative analysis. The primary goal was to assess the diagnostic performance of PF-LAM relative to the lab-based uLAM assay, EclLAM, which utilizes the same antibody pair as PF-LAM and uses MRS as a reference test.

The banked urine samples were also characterized by plasmonic-linked immunosorbent assay (PFLISA-LAM), which is an ultra-sensitive tool to detect urinary LAM, demonstrated in a recent study (27).

### Biobanked urine samples

Comprehensive microbiological data (sputum Xpert MTB/RIF, smear microscopy, culture) and clinical information were collected at enrollment and during follow-up and linked to the corresponding urine specimens. Each urine sample was aliquoted and stored in individual tubes. For both studies, the FIND TB team requested the biospecimen bank to randomly select samples representing a spectrum of microbiological results, ensuring balanced representation of TB-positive and TB-negative cases. All samples were obtained from adults between 2016 and 2021 and had been stored for 4–9 years. TB-positive cases in both studies were confirmed as pulmonary TB. The frozen aliquots (3–5 mL) of each sample were sent to PATH, Brightest Bio. After receiving the frozen aliquots, the samples were thawed at room temperature and then divided into smaller volume (~0.1–0.5 mL). After aliquoting, the samples were then stored in the freezer (−80°C).

### Assay procedures

#### PF-LAM

The frozen urine samples were thawed by placing them in a 60°C oven for 10 min, followed by an additional incubation step for 30 min at room temperature. After thawing, 200 µL of each sample was transferred into a reagent tube containing lyophilized reagent powder. After mixing, the samples were incubated for 40 min. Then, 65 µL of the mixture was added to the test cassette. Following a 20-min running of the test, the cassette was then inserted into the Brightest Bio fluorescent LFA reader for scanning and result analysis. The diagnostic accuracy panel was also tested by PF-LAM using different assay times (30 min, 1 h, 2 h).

#### AlereLAM

Testing was performed per the manufacturer’s instructions using the same aliquots as PF-LAM. Two operators independently graded each test against the manufacturer’s card, and optical images were recorded for documentation. Discordant results were resolved by analyzing the images with ImageJ to reach a final consensus.

#### EclLAM

Blinded EclLAM testing at both MSD and PATH was conducted following a previously established assay protocol, without the use of a preconcentration step (24).

### PF-LAM assay design

The PF-LAM assay consists of two parts: reagent powder and LFA cassette. The reagent powder consists of optimized buffer and PF-conjugated with detection antibody (A194-01, HPAB-M0560-YC, Creative Biolabs, USA) in lyophilized powder form (Fig. S4). The LFA cassette consists of a sample pad, a nitrocellulose membrane, an absorbent pad, and a custom cassette. The sample pad was treated with surfactant. The nitrocellulose membrane contains a test line with S4-20 (Otsuka Pharmaceuticals, Japan) and a control line with anti-human IgG (109-005-003, RRID: AB_2337532, Jackson ImmunoResearch Laboratories, USA). Since the Anti-LAM detection antibody (A194-01) is a human monoclonal antibody, the control line would be consistently detectable across all tests, while the test line should be only detected when the LAM is present. Both the detection antibody (A194-01) and the capture antibody (S4-20) have been studied and validated previously (8, 23, 28).

The PF-LAM is scanned and analyzed under a fluorescent LFA reader developed by Brightest Bio. The LFA reader uses a built-in algorithm to determine both the fluorescent intensity and the SNR at the test line, ensuring accurate result interpretation. The test outcomes are interpreted based on SNR.

### PF-LAM assay evaluation

#### Detection limit

The LOB was evaluated by measuring 50 TB-negative urine samples from the preclinical panel. The LOB was calculated as follows: mean_blank_ + 1.645 (SD_blank_). For the limit of detection (LOD) evaluation, purified cultured LAM (cLAM, BEI, H37Rv, NR-14848) was spiked into TB-negative urine and serially diluted to achieve a panel with 10 concentrations (0–1,000 pg/mL). This panel was measured using PF-LAM under different incubation times (10, 40, 100 min) and the same assay running time of 20 min, leading to total assay times of 30 min, 1 h, and 2 h, respectively. This panel was also measured using PF-LAM by three replicates per concentration with two different lots. LOD is defined as the lowest concentration of LAM that can be measured consistently.

#### Precision and stability

A panel comprising TB-negative urine, 5 pg/mL cLAM-spiked urine, 10 pg/mL cLAM-spiked urine, and 20 pg/mL cLAM-spiked urine was tested in five replicates, using the PF-LAM to assess the repeatability and reproducibility across negative, weakly reactive, medium reactive samples. A dose-response panel, including TB-negative urine, cLAM spiked samples ranging from 3.9 to 62.5 pg/mL, was evaluated using five different lots of PF-LAM kits. One of the tests was scanned using four different LFA readers to assess inter-reader variability. To evaluate the stability, PF-LAM kits sealed in pouches were stored at 28°C for 12 weeks. Each week, the PF-LAM was tested using negative (H_2_O) and positive (1,000 pg/mL cLAM) controls. In addition, a panel of TB-negative urine, 5 pg/mL cLAM-spiked, and 10 pg/mL cLAM-spiked urine samples were tested every 4 weeks with the stored kits. Finally, the diagnostic accuracy panel was tested on different days using three different lots of PF-LAM kits to evaluate consistency in diagnostic performance. All the above tests were performed using 1-h assay time.

### Statistical analysis

We calculated descriptive statistics for the demographic and clinical variables of participants with and without TB in both studies. We also calculated and summarized descriptive statistics for the uLAM concentration in urine samples measured by PFLISA and EclLAM (27).

In the preclinical study, ROC analysis determined the optimal PF-LAM cut-off at an SNR of 1.012 (corresponding to ~10 pg/mL of uLAM in urine), achieving 98% specificity and meeting WHO TPP requirements versus culture (2, 29). Point estimates and 95% Wilson CIs were calculated for the sensitivity, specificity for PF-LAM and AlereLAM versus MRS.

For the diagnostic accuracy study, ROC analysis established optimal EclLAM cut-offs based on back-calculated uLAM concentrations: 58 pg/mL for MSD data and 49 pg/mL for PATH, each achieving 95% specificity. Point estimates and 95% Wilson CIs were calculated for PF-LAM, EclLAM, and AlereLAM, using MRS as well as smear, culture, and Xpert as comparators.

All statistical analyses were conducted in GraphPad Prism 10.

## SUPPLEMENTAL MATERIAL

Supplemental figures and tables spectrum.03042-25-s0001.docx Figures S1 to S4 and Tables S1 to S13.

ASM does not own the copyrights to Supplemental Material that may be linked to, or accessed through, an article. The authors have granted ASM a non-exclusive, world-wide license to publish the Supplemental Material files. Please contact the corresponding author directly for reuse.
