# Supplementary material for: Rapid urinary lipoarabinomannan test with laboratory-level sensitivity for tuberculosis detection: a performance evaluation
Source: Microbiol Spectr. 2025 Dec 31;14(2):e03042-25. doi: 10.1128/spectrum.03042-25 (PMC12889118; doi:10.1128/spectrum.03042-25)
Supplement: Supplemental figures and tables — Figures S1 to S4 and Tables S1 to S13. [file spectrum.03042-25-s0001.docx]

**Supplementary Figures**

**Figure S1.** ROC curve of PF-LAM assay performance on the preclinical panel. The cut-off was selected at 1.012 so that the specificity reached 98%, surpassing WHO’s TPP specificity requirement of 98% versus culture.

**Figure S2.** ROC curves of EclLAM conducted at different sites (MSD and PATH) on the diagnostic accuracy panel.

**Figure S3.** ROC curves of PF-LAM with different assay time on the diagnostic accuracy panel.

**
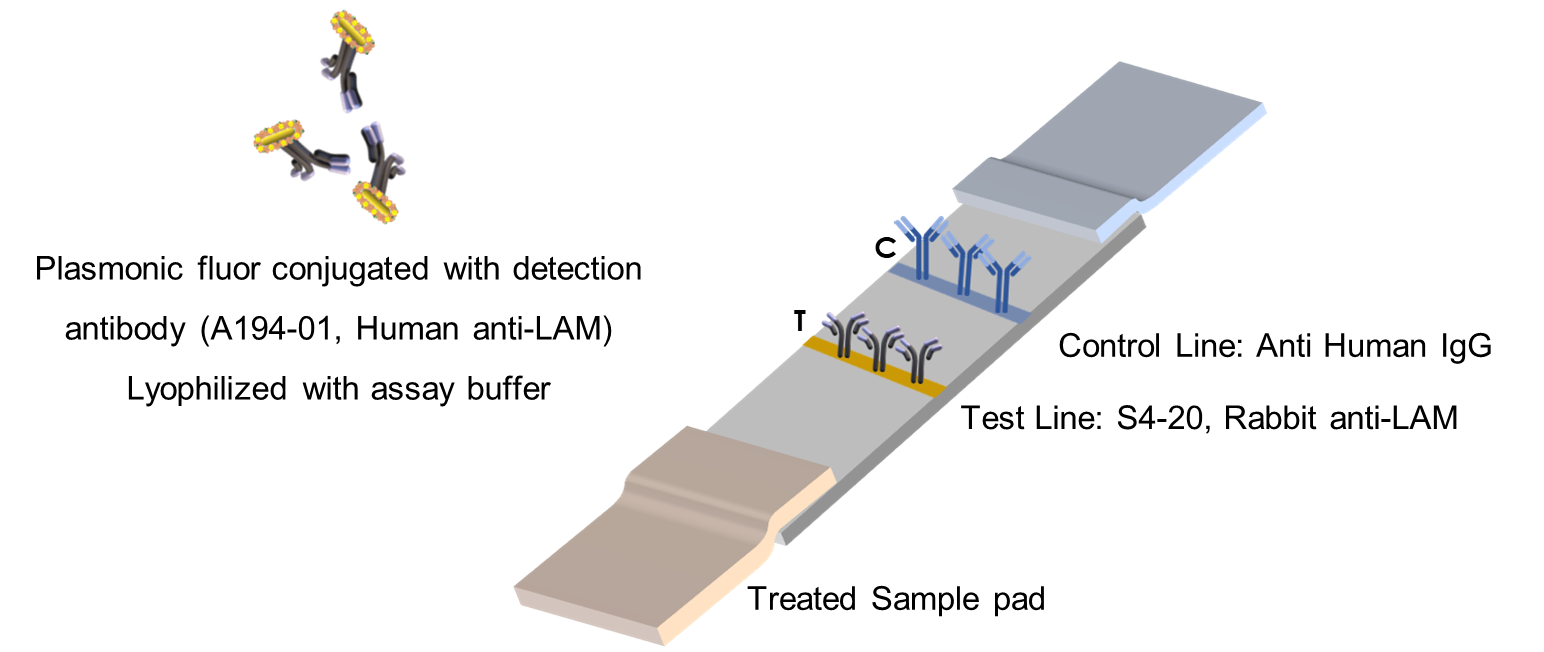
**

**Figure S4.** Schematic showing the assay design of plasmonic fluor-enhanced lateral flow assay for LAM (PF-LAM).

**Supplementary tables**

|  | **30min assay** | | **1hr assay** | | **2hr assay** | |
| --- | --- | --- | --- | --- | --- | --- |
| **Concentration**  **(pg/mL)** | **FL** | **SD** | **FL** | **SD** | **FL** | **SD** |
| **Blank** | 1050 | 16 | 1039 | 16 | 1007 | 16 |
| **2** | 1101 | 135 | 1319 | 157 | 1647 | 183 |
| **5** | 1332 | 80 | 2688 | 250 | 4500 | 167 |
| **10** | 2918 | 788 | 5294 | 806 | 12000 | 1287 |
| **25** | 7386 | 1568 | 14221 | 2553 | 28862 | 4735 |
| **50** | 14077 | 3135 | 37371 | 4243 | 65409 | 7311 |
| **100** | 35950 | 2122 | 89806 | 11546 | 144407 | 15367 |
| **250** | 85403 | 4349 | 256397 | 13772 | 409652 | 29454 |
| **500** | 170806 | 6523 | 452794 | 20658 | 819304 | 44182 |
| **1000** | 411269 | 36935 | 766758 | 69142 | 1216270 | 185525 |
| **Table S1.** Fluorescent intensity results generated by PF-LAM using the analytical sensitivity panel with 9 dilutions in 3 replicates. FL=fluorescent intensity. SD=standard deviation. | | | | | | |

|  | **30min assay** | | **1hr assay** | | **2hr assay** | |
| --- | --- | --- | --- | --- | --- | --- |
| **Concentration (pg/mL)** | **SNR** | **SD** | **SNR** | **SD** | **SNR** | **SD** |
| **Blank** | 0.998 | 0.003 | 0.999 | 0.003 | 0.998 | 0.001 |
| **2** | 1.001 | 0.000 | 1.002 | 0.000 | 1.003 | 0.000 |
| **5** | 1.002 | 0.000 | 1.005 | 0.000 | 1.012 | 0.001 |
| **10** | 1.006 | 0.001 | 1.013 | 0.001 | 1.028 | 0.001 |
| **25** | 1.012 | 0.001 | 1.027 | 0.002 | 1.051 | 0.001 |
| **50** | 1.024 | 0.001 | 1.065 | 0.006 | 1.109 | 0.003 |
| **100** | 1.066 | 0.004 | 1.133 | 0.011 | 1.237 | 0.028 |
| **250** | 1.123 | 0.001 | 1.264 | 0.020 | 1.415 | 0.084 |
| **500** | 1.266 | 0.012 | 1.475 | 0.015 | 1.959 | 0.044 |
| **1000** | 1.537 | 0.009 | 1.864 | 0.007 | 2.364 | 0.171 |
| **Table S2.** SNR results generated by PF-LAM using the analytical sensitivity panel with 9 dilutions in 3 replicates. SNR=signal-to-noise. SD=standard deviation. | | | | | | |

|  | **Lot 2443-01** | **Lot 2445-01** | **Lot 2445-02** | **Lot 2515-01** | **Lot 2521-02** |
| --- | --- | --- | --- | --- | --- |
| Blank | 1.002 | 1.002 | 1.001 | 1.000 | 0.998 |
| 3.9 pg/mL | 1.007 | 1.006 | 1.006 | 1.004 | 1.005 |
| 7.8 pg/mL | 1.010 | 1.011 | 1.009 | 1.009 | 1.008 |
| 15.63 pg/mL | 1.027 | 1.019 | 1.015 | 1.013 | 1.017 |
| 31.25 pg/mL | 1.044 | 1.052 | 1.031 | 1.023 | 1.045 |
| 62.5 pg/mL | 1.081 | 1.078 | 1.071 | 1.054 | 1.072 |
| **Table S3.** Signal-to-noise ratio (SNR) generated by one LFA reader. PF-LAM was tested using purified LAM spiked in negative urine (1hr assay) with 5 different lots of PF-LAM test kits. | | | | | |

| **SNR** | **Reader 1** | **Reader 2** | **Reader 3** | **Reader 4** |
| --- | --- | --- | --- | --- |
| Blank | 1.005 | 0.999 | 0.998 | 1.000 |
| 3.9 pg/mL | 1.011 | 1.005 | 1.002 | 1.006 |
| 7.8 pg/mL | 1.012 | 1.008 | 1.007 | 1.006 |
| 15.63 pg/mL | 1.023 | 1.018 | 1.018 | 1.016 |
| 31.25 pg/mL | 1.040 | 1.031 | 1.041 | 1.034 |
| 62.5 pg/mL | 1.069 | 1.059 | 1.067 | 1.058 |
|  |  |  |  |  |
| **Test result** | **Reader 1** | **Reader 2** | **Reader 3** | **Reader 4** |
| Blank | Negative | Negative | Negative | Negative |
| 3.9 pg/mL | Negative | Negative | Negative | Negative |
| 7.8 pg/mL | Positive | Negative | Negative | Negative |
| 15.63 pg/mL | Positive | Positive | Positive | Positive |
| 31.25 pg/mL | Positive | Positive | Positive | Positive |
| 62.5 pg/mL | Positive | Positive | Positive | Positive |
| **Table S4.** Signal-to-noise ratio (SNR) and test results generated by different fluorescent readers. The SNR cut-off was set at 1.012. PF-LAM was tested using purified LAM spiked in negative urine (1hr assay). | | | | |

| **Cut-off (pg/mL)** | **Sensitivity%** | **95% CI** | **Specificity%** | **95% CI** |
| --- | --- | --- | --- | --- |
| > 1.010 | 35.32 | 29.05% to 42.15% | 94.95 | 90.95% to 97.23% |
| > 1.010 | 35.32 | 29.05% to 42.15% | 95.45 | 91.59% to 97.59% |
| > 1.010 | 35.32 | 29.05% to 42.15% | 95.96 | 92.23% to 97.94% |
| > 1.010 | 35.32 | 29.05% to 42.15% | 96.46 | 92.88% to 98.28% |
| > 1.011 | 35.32 | 29.05% to 42.15% | 96.97 | 93.55% to 98.60% |
| > 1.011 | 34.83 | 28.58% to 41.64% | 97.98 | 94.92% to 99.21% |
| **> 1.012** | **33.83** | **28.58% to 41.64%** | **98.48** | **95.64% to 99.59%** |
| > 1.012 | 33.33 | 28.11% to 41.13% | 98.48 | 95.64% to 99.59% |
| > 1.012 | 32.84 | 27.65% to 40.62% | 98.48 | 95.64% to 99.59% |
| > 1.012 | 32.34 | 27.18% to 40.11% | 98.48 | 95.64% to 99.59% |
| > 1.013 | 31.84 | 26.72% to 39.60% | 98.48 | 95.64% to 99.59% |
| > 1.013 | 31.34 | 26.26% to 39.08% | 98.48 | 95.64% to 99.59% |
| > 1.013 | 30.85 | 25.79% to 38.57% | 98.48 | 95.64% to 99.59% |
| > 1.013 | 30.35 | 25.33% to 38.06% | 98.48 | 95.64% to 99.59% |
| > 1.013 | 29.85 | 24.87% to 37.54% | 98.48 | 95.64% to 99.59% |
| > 1.013 | 29.35 | 24.41% to 37.02% | 98.48 | 95.64% to 99.59% |
| > 1.013 | 28.86 | 23.95% to 36.51% | 98.48 | 95.64% to 99.59% |
| > 1.013 | 28.36 | 23.49% to 35.99% | 98.48 | 95.64% to 99.59% |
| > 1.013 | 27.86 | 23.03% to 35.47% | 98.48 | 95.64% to 99.59% |
| > 1.013 | 27.36 | 22.58% to 34.95% | 98.48 | 95.64% to 99.59% |
| > 1.014 | 27.36 | 22.12% to 34.43% | 98.48 | 95.64% to 99.59% |
| > 1.014 | 26.87 | 21.67% to 33.91% | 98.48 | 95.64% to 99.59% |
| > 1.014 | 26.87 | 21.67% to 33.91% | 98.99 | 96.39% to 99.82% |
| > 1.014 | 26.37 | 21.21% to 33.39% | 98.99 | 96.39% to 99.82% |
| > 1.015 | 25.87 | 21.21% to 33.39% | 99.49 | 97.20% to 99.97% |
| > 1.015 | 25.37 | 20.76% to 32.86% | 99.49 | 97.20% to 99.97% |
| > 1.016 | 24.88 | 20.31% to 32.34% | 99.49 | 97.20% to 99.97% |
| > 1.016 | 24.38 | 19.86% to 31.81% | 99.49 | 97.20% to 99.97% |
| > 1.017 | 23.88 | 19.41% to 31.29% | 99.49 | 97.20% to 99.97% |
| > 1.018 | 23.38 | 18.96% to 30.76% | 99.49 | 97.20% to 99.97% |
| > 1.018 | 22.89 | 18.51% to 30.23% | 99.49 | 97.20% to 99.97% |
| > 1.020 | 22.89 | 17.62% to 29.17% | 100 | 98.10% to 100.0% |

**Table S5.** ROC analysis data for PF-LAM in the preclinical study versus culture. The data is selected from the where the specificity falls between 95%-99%.

| **CD4 Count**  **(cells/µl)** | **Assay** | **Sensitivity [n/n, (95% CI)]** | **Specificity [n/n, (95% CI)]** |
| --- | --- | --- | --- |
| **0-200** | **AlereLAM** | 2/5, 40% (5-85) | 3/3, 100% (29-100) |
|  | **PF-LAM** | 4/5, 80% (28-99) | 3/3, 100% (29-100) |
| **>200** | **AlereLAM** | 2/7, 29% (4-71) | 8/8, 100% (63-100 |
|  | **PF-LAM** | 6/7, 86% (42-99) | 8/8, 100% (63-100) |
| **Unknown** | **AlereLAM** | 1/17, 6% (0-29) | 10/10, 100% (69-100) |
|  | **PF-LAM** | 8/17, 47% (23-72) | 9/10, 90% (56-99) |
| **Table S6.** Diagnostic accuracy of PF-LAM and AlereLAM among HIV-positive cases stratified by CD4 count in the preclinical study | | | |

|  |  | **Median** | **IQR** | **Mean** | **SD** | **Min** | **Max** |
| --- | --- | --- | --- | --- | --- | --- | --- |
| **EclLAM**  **(MSD)** | TB-negative N=(44) | 0 | 0-6 | 9 | 25 | 0 | 134 |
|  | S-C+ (N=5) | 0 | 0-12 | 5 | 8 | 0 | 19 |
|  | S+C+ (N=26) | 71 | 8-139 | 116 | 187 | 0 | 836 |
|  | C+ (N=31) | 25 | 0-119 | 98 | 176 | 0 | 836 |
|  | C+Xpert+ (N=33) | 19 | 0-118 | 92 | 171 | 0 | 836 |
| **EclLAM (PATH)** | TB-negative N=(44) | 0 | 0-0 | 11 | 53 | 0 | 344 |
|  | S-C+ (N=5) | 0 | 0-0 | 0 | 0 | 0 | 0 |
|  | S+C+ (N=26) | 61 | 16-165 | 161 | 224 | 0 | 661 |
|  | C+ (N=31) | 42 | 0-152 | 135 | 213 | 0 | 661 |
|  | C+Xpert+ (N=33) | 34 | 0-150 | 126 | 209 | 0 | 661 |
| **PFLISA-LAM** | TB-negative N=(44) | 0 | 0-0 | 1 | 7 | 0 | 50 |
|  | S-C+ (N=5) | 0 | 0-0 | 0 | 0 | 0 | 1 |
|  | S+C+ (N=26) | 30 | 7-80 | 60 | 83 | 0 | 304 |
|  | C+ (N=31) | 19 | 2-60 | 50 | 79 | 0 | 304 |
|  | C+Xpert+ (N=33) | 16 | 0-56 | 47 | 77 | 0 | 304 |
| SD, Standard deviation; IQR, Interquartile Range; Min, Minimum; Max, Maximum; | | | | | | | |
| **Table S7.** LAM concentration (pg/mL) in urine measured by EclLAM (MSD and PATH) and PFLISA-LAM among TB-negative and TB-positive cases in the diagnostic accuracy study. Both platforms provided consistent trends: S+C+ samples showed higher uLAM concentrations than C+ and S–C+ samples, while TB-negative samples exhibited very low or negligible uLAM levels. S-C- = smear-negative and culture negative. S-C+ = smear-negative and culture-positive. S+C+ = smear-positive and culture-positive. C+ = culture-positive. | | | | | | | |

|  | | **Preclinical panel (N=201)** | | **Diagnostic accuracy panel (N=33)** | |
| --- | --- | --- | --- | --- | --- |
|  |  | **N (%)** | **Median LAM concentration (IQR) (pg/mL)** | **N (%)** | **Median LAM concentration (IQR) (pg/mL)** |
| **Location** | Vietnam | 82 (41%) | 2 (1-8) | 4 (12%) | 62 (11-189) |
|  | Peru | 57 28%) | 6 (1-30) | 20 (61%) | 14 (2-57) |
|  | South Africa | 55 (27%) | 4 (0-18) | 9 (27%) | 19 (0-33) |
| **HIV status** | HIV - | 172 (86%) | 3 (1-15) | 26 (79%) | 14 (1-54) |
|  | HIV+ | 29 (14%) | 10 (1-28) | 7 (21%) | 29 (1-100) |
| **Enrollment time** | 2016-2019 | 180 (90%) | 3 (1-14) | 17 (52%) | 2 (0-33) |
|  | 2020-2021 | 21 (10%) | 17 (5-76) | 16 (48%) | 32 (13-96) |
| **Xpert positivity** | Xpert-negative | 83 (41%) | 2 (1-9) | 4 (12%) | 0 (0-1) |
|  | Xpert-positive | 118 (59%) | 5 (1-27) | 29 (88%) | 21 (4-68) |
| **Table S8.** Comparison of the median concentration (measured by PFLISA-LAM) of TB-positive cases in the preclinical and diagnostic accuracy panel, stratified by geography, HIV status, and enrollment period. Samples collected in 2020–2021 within the diagnostic panel had a markedly higher median concentration (32 pg/mL; IQR 13–96) than those from 2016–2019 (2 pg/mL; IQR 0–33). This recent period accounted for 48% of the diagnostic panel but only 10% of the preclinical panel. In the preclinical panel, the median uLAM concentration detected by PFLISA-LAM in MRS TB-positive cases was 3 pg/mL (IQR 1–17). This value increased to 16 pg/mL (IQR 0–56) in the diagnostic accuracy panel. Correspondingly, PF-LAM showed greater sensitivity in the diagnostic panel than in the preclinical one (58% vs. 33.8%). The primary factor driving this difference appears to be the higher proportion of samples collected during 2020–2021 in the diagnostic panel (48% vs. 10%). Additionally, the diagnostic panel included more samples from HIV-positive and Xpert-positive patients (21% vs. 14% and 88% vs. 59%, respectively), which may have further contributed to the elevated LAM levels. Despite differences in patient characteristics or enrollment periods between the two panels, these factors do not influence the within-panel comparison between EclLAM and PF-LAM measurements. | | | | | |

|  | **Sensitivity [n/n, (95% CI)]** | | | | | **Specificity [n/n, (95% CI)]** |
| --- | --- | --- | --- | --- | --- | --- |
|  | **S-C-Xpert+ (N=2)** | **S-C+**  **(N = 5)** | **S+C+**  **(N = 26)** | **C+**  **(N = 31)** | **MRS+**  **(N = 33)** | **MRS-**  **(N = 44)** |
| **AlereLAM** | 0/2 | 0/5 | 3/26,  12% (2-30) | 3/31,  10% (3-26) | 3/33,  9% (3-24) | 43/44,  98% (88-100) |
| **EclLAM (PATH)** | 0/2 | 0/5 | 14/26,  54% (34-73) | 14/31,  45% (28-64) | 14/33,  42% (27-59) | 42/44,  95% (85-99) |
| **EclLAM (MSD)** | 0/2 | 0/5 | 14/26,  54% (34-73) | 14/31,  45% (28-64) | 14/33,  42% (27-59) | 42/44,  95% (85-99) |
| **PF-LAM** | 0/2 | 0/5 | 19/26,  73% (52-88) | 19/31,  61% (42-78) | 19/33,  58% (41-73) | 43/44,  98% (88-100) |
| S, smear microscopy; C, culture. Xpert, sputum Xpert MTB/RIF | | | | | | |
| **Table S9.** Diagnostic accuracy of PF-LAM and EclLAM versus smear, culture and Xpert results. S-C- = smear-negative and culture negative. S-C+ = smear-negative and culture-positive. S+C+ = smear-positive and culture-positive. C+ = culture-positive. | | | | | | |

|  | **Sensitivity [n/n, (95% CI)]** | | **Specificity [n/n, (95% CI)]** | |
| --- | --- | --- | --- | --- |
|  | **HIV+ (N=7)** | **HIV- (N=26)** | **HIV+ (N=12)** | **HIV- (N=32)** |
| **AlereLAM** | 2/7, 29% (4-71) | 1/26, 4% (0-20) | 12/12, 100% (74-100) | 31/32, 97% (84-100) |
| **EclLAM (PATH)** | 3/7, 43% (12-80) | 11/26, 42% (24-63) | 11/12, 92% (60-100) | 31/32, 97% (82-100) |
| **EclLAM (MSD)** | 4/7, 57% (20-88) | 10/26, 38% (21-59) | 11/12, 92% (60-100) | 31/32, 97% (82-100) |
| **PF-LAM** | 5/7, 71% (29-96) | 14/26, 54% (33-73) | 11/12, 92% (60-100) | 32/32, 100% (90-100) |
| **Table S10.** Diagnostic accuracy of PF-LAM, EclLAM stratified by HIV status versus MRS. | | | | |

| **CD4 Count (cells/µl)** | **Assay** | **Sensitivity (n/n)** | **Specificity (n/n)** |
| --- | --- | --- | --- |
| **0-200** | **AlereLAM** | 1/2 | 3/3 |
|  | **EclLAM (PATH)** | 2/2 | 2/3 |
|  | **EclLAM (MSD)** | 2/2 | 2/3 |
|  | **PF-LAM** | 2/2 | 2/3 |
| **>200** | **AlereLAM** | 0/4 | 8/8 |
|  | **EclLAM (PATH)** | 0/4 | 8/8 |
|  | **EclLAM (MSD)** | 1/4 | 8/8 |
|  | **PF-LAM** | 2/4 | 8/8 |
| **Unknown** | **AlereLAM** | 1/1 | 1/1 |
|  | **EclLAM (PATH)** | 1/1 | 1/1 |
|  | **EclLAM (MSD)** | 1/1 | 1/1 |
|  | **PF-LAM** | 1/1 | 1/1 |
| **Table S11.** Diagnostic accuracy of AlereLAM, PF-LAM and EclLAM among HIV-positive cases stratified by CD4 count in the diagnostic study. | | | |

|  | **Sensitivity [n/n, (95% CI)]** | | **Specificity [n/n, (95% CI)]** | |
| --- | --- | --- | --- | --- |
|  | **S+C+**  **(N = 26)** | **C+**  **(N = 31)** | **MRS+**  **(N = 33)** | **MRS-**  **( N = 44)** |
| **30-minute** | 14/26,  54% (34-73) | 14/31,  45% (28-64) | 14/33,  42% (27-59) | 43/44,  98% (88-100) |
| **1-hour** | 19/26,  73% (52-88) | 19/31,  61% (42-78) | 19/33,  58% (41-73) | 43/44,  98% (88-100) |
| **2-hour** | 20/26,  77% (56-91) | 20/31,  65% (45-81) | 20/33,  61% (44-75) | 43/44,  98% (88-100) |
| **Table S12.** **Diagnostic accuracy comparison of PF-LAM with different assay time.** 30-minute = 10-minute incubation+20-minute running; 1-hour = 40-minute incubation + 20-minute running; 2-hour = 100-minute incubation + 20-minute running. S=smear microscopy. C=culture. Xpert=sputum Xpert MTB/RIF. | | | | |

| **LAM assays** | **Assay type** | **Core Technology** | **Compared to AlereLAM**  **vs MRS** | **Compared to AlereLAM**  **vs HIV+** | **Compared to AlereLAM**  **vs HIV-** |
| --- | --- | --- | --- | --- | --- |
| FujiLAM  (Fujifilm) | 1hr RDT | Silver amplification | / | 1.7X^1^ | 4.9X^1^ |
| FujiLAM 2 (Fujifilm) | 1hr RDT | Silver amplification | / | 0.8X^2^**,** 0.7X^3^ | / |
| BP-LAM (Biopromic) | 1hr RDT | Magnetic bead sample treatment/concentration | 3.1X^4^ | 2.5X^4^ | 3.3X^4^ |
| EclLAM (Meso Scale Diagnostic) | 2hr lab-test | Electrochemiluminescence | 4.7X | / | 6.2X^1^ |
| **PF-LAM**  **(this work)** | 1hr RDT | Plasmonic fluor | 6.2X | 3.6X | 8.3X |
| EclLAM, FujiLAM, FujiLAM2 and PF-LAM utilize the same antibody pair: S4-20/A194;  MRS= microbiology reference standard. | | | | | |
| **Table S13.** Diagnostic performance comparison between existing urinary LAM assays. Because studies differ in design and populations, we report each assay’s relative yield vs. AlereLAM—the fold-increase in TB cases detected compared with AlereLAM (reference = 1.0). | | | | | |

**References**

1. Broger, T.; Nicol, M. P.; Sigal, G. B.; Gotuzzo, E.; Zimmer, A. J.; Surtie, S.; Caceres-Nakiche, T.; Mantsoki, A.; Reipold, E. I.; Székely, R.; Tsionsky, M.; van Heerden, J.; Plisova, T.; Chikamatsu, K.; Lowary, T. L.; Pinter, A.; Mitarai, S.; Moreau, E.; Schumacher, S. G.; Denkinger, C. M., Diagnostic accuracy of 3 urine lipoarabinomannan tuberculosis assays in HIV-negative outpatients. *The Journal of Clinical Investigation* **2020,** *130* (11), 5756-5764.

2. Ellis, J.; Dai, B.; Kabahubya, M.; Hale, G.; Mande, E.; Katende, G.; Kagimu, E.; Gakuru, J.; Ndyetukira, J. F.; Tukundane, A.; Adzemovic, T.; Nsangi, L. J.; Jarvis, J. N.; Bahr, N. C.; Cresswell, F. V.; Meya, D. B.; Boulware, D. R., Diagnostic accuracy of the re-engineered urinary FujiLAM2 assay amongst hospitalized adults with advanced HIV disease. *AIDS* **2025,** *39* (10), 1334-1343.

3. Adzemovic, T.; Nalintya, E.; Fieberg, A.; Mande, E.; George, K.; Yueh, S.; Namakula, O.; Nerima, P.; Dai, B.; Meya, D. B.; Boulware, D. R.; Rajasingham, R.; team, o. b. o. t. E. s., Diagnostic Accuracy of the Updated FujiLAM II Assay to Detect Tuberculosis in Outpatients With Advanced HIV Disease. *Clinical Infectious Diseases* **2025**.

4. Beston Hamasur, A. O. O., James Sserubiri et al. A new rapid lipoarabinomannan urine assay for tuberculosis: a two-centre diagnostic accuracy evaluation in outpatients with and without HIV, 19 February 2025, PREPRINT (Version 2) available at Research Square [<https://doi.org/10.21203/rs.3.rs-5386988/v2>].
